# Functional Organization of Sequence Motifs in Diverse Transit Peptides of Chloroplast Proteins

**DOI:** 10.3389/fphys.2021.795156

**Published:** 2021-11-22

**Authors:** Jinseung Jeong, Inhwan Hwang, Dong Wook Lee

**Affiliations:** ^1^Department of Integrative Food, Bioscience and Biotechnology, Chonnam National University, Gwangju, South Korea; ^2^Department of Life Sciences, Pohang University of Science and Technology, Pohang, South Korea; ^3^Department of Bioenergy Science and Technology, Chonnam National University, Gwangju, South Korea

**Keywords:** chloroplast, transit peptide, sequence motif, protein targeting, protein translocation

## Abstract

Although the chloroplasts in plants are characterized by an inherent genome, the chloroplast proteome is composed of proteins encoded by not only the chloroplast genome but also the nuclear genome. Nuclear-encoded chloroplast proteins are synthesized on cytosolic ribosomes and post-translationally targeted to the chloroplasts. In the latter process, an N-terminal cleavable transit peptide serves as a targeting signal required for the import of nuclear-encoded chloroplast interior proteins. This import process is mediated *via* an interaction between the sequence motifs in transit peptides and the components of the TOC/TIC (translocon at the outer/inner envelope of chloroplasts) translocons. Despite a considerable diversity in primary structures, several common features have been identified among transit peptides, including N-terminal moderate hydrophobicity, multiple proline residues dispersed throughout the transit peptide, preferential usage of basic residues over acidic residues, and an absence of N-terminal arginine residues. In this review, we will recapitulate and discuss recent progress in our current understanding of the functional organization of sequence elements commonly present in diverse transit peptides, which are essential for the multi-step import of chloroplast proteins.

## Introduction

The chloroplast, a type of plastid, is an organelle uniquely present in plants and is believed to have been derived *via* the endosymbiosis of ancient cyanobacteria (Dyall et al., 2004; Zimorski et al., 2014; Lee and Hwang, 2021). During the subsequent evolution of chloroplasts as subcellular organelles, more than 90% of the original cyanobacterial genes were transferred to the host nuclear genome (Ponce-Toledo et al., 2019). Consequently, both chloroplastic and nuclear genome systems now contribute to assembly of the chloroplast proteome in plants. Most of the nuclear-encoded chloroplast proteins are synthesized on cytosolic ribosomes and targeted to chloroplasts post-translationally (Inaba and Schnell, 2008; Li and Chiu, 2010; Lee et al., 2013). These nuclear-encoded chloroplast proteins can be broadly categorized based on their sub-organellar locations. Proteins localized to the outer membrane of chloroplasts are primarily inserted directly into the outer membrane from the cytosol, with a few exceptions (Lee et al., 2014, 2017; Day et al., 2019; Gross et al., 2021). However, most chloroplast interior proteins, which are localized to the inner membrane, stroma, or thylakoids, are imported into chloroplasts in a process mediated by the TOC/TIC (translocon at the outer/inner envelope of chloroplasts) translocons (Li and Chiu, 2010; Richardson et al., 2017, 2018). The chloroplast interior proteins are characterized by the presence of N-terminal cleavable targeting signals, called transit peptides (Bruce, 2000; Lee and Hwang, 2018), one of the hallmarks of which is the considerable diversity in their primary structure (Bruce, 2000; Lee et al., 2008, 2015; Li and Teng, 2013). It has previously been proposed that these diverse transit peptides might have been generated *via* the selective assembly of short sequence motifs that are critical for interactions with the components of the TOC/TIC translocons (Li and Teng, 2013; Lee et al., 2015; Lee and Hwang, 2018). Indeed, it has been established that different transit peptides contain distinct sequence motifs that play vital roles during the multi-step import process (Lee et al., 2006, 2008; Li and Teng, 2013; Lee and Hwang, 2018). Moreover, a previous study indicated that diverse transit peptides can be classified into multiple subgroups based on their sequence motifs (Lee et al., 2008).

Conversely, in addition to the inherent diversity of transit peptides, recent studies have revealed the presence of common sequence elements among different chloroplast transit peptides. In this review, we will discuss how these common features contribute to the appropriate functioning of transit peptides and provide an overview of the organization of these sequence features in transit peptides.

## Sequence Motifs in the Rubisco Small Subunit Transit Peptide as a Model System

Ribulose-1,5-bisphosphate carboxylase/oxygenase (Rubisco) is acknowledged to be the most abundant protein in nature, accounting for approximately 30% of total leaf proteins (Jensen, 2000). As an enzyme that is prominently involved in CO_2_ fixation, Rubisco plays a central role in photosynthesis. The Rubisco complex comprises the Rubisco small subunit (RbcS) and Rubisco large subunit. Within the chloroplast stroma, the Rubisco large subunit produced in the chloroplast is assembled with the RbcS that is encoded in the nuclear genome and imported into the chloroplasts *via* the TOC/TIC translocons (Lee et al., 2006; Chotewutmontri et al., 2012; Holbrook et al., 2016). Given the abundance and essential roles of the RbcS in chloroplasts, the mechanisms underlying the import of RbcS have been extensively studied in several species as a representative chloroplast cargo protein, thereby providing a new perspective regarding our understanding of chloroplast biogenesis (Karlin-Neumann and Tobin, 1986; Reiss et al., 1987; Becker et al., 2004; Smith et al., 2004; Lee et al., 2006, 2015; Chotewutmontri et al., 2012; Holbrook et al., 2016; Richardson et al., 2018; Chen et al., 2019).

**Figure 1:**
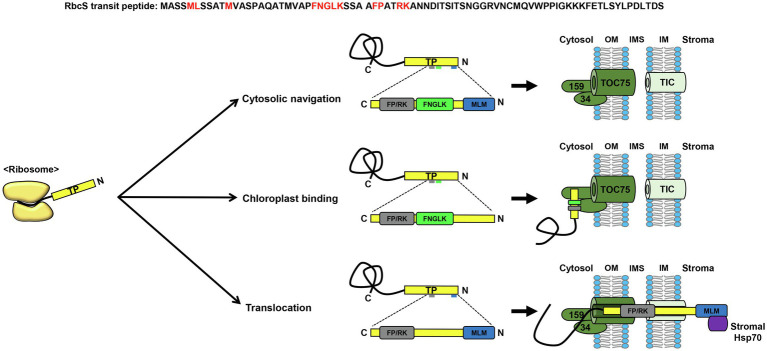
Sequence motifs in the Rubisco small subunit transit peptide. Among the sequence motifs in the Rubisco small subunit (RbcS) transit peptide, the motifs, MLM, FNGLK, and FP/RK have been extensively characterized. The MLM motif is vital not only for cytosolic navigation, but also for translocation across chloroplast membranes, by interacting with stromal Hsp70. The FNGLK and FP/RK motifs have been demonstrated to mediate chloroplast binding *via* interactions with Toc34. The segment containing the FP/RK motif lies in close proximity to the TOC/TIC translocons during preprotein translocation, which may be essential for the efficient translocation of RbcS. TP, transit peptide; OM, outer membrane; IMS, intermembrane space; IM, inner membrane.

Early attempts to elucidate the organization of the RbcS transit peptide led to the conclusion that it contains multiple domains that play important roles during protein import into chloroplasts (Karlin-Neumann and Tobin, 1986; von Heijne et al., 1989; Bruce, 2001; Becker et al., 2004). Subsequently, extensive mutagenesis analysis of the *Arabidopsis* RbcS transit peptide revealed the presence of distinct sequence motifs, each of which proved to be essential for the correct cytosolic navigation, chloroplast binding, or translocation of chloroplast preproteins across the envelope membranes (Lee et al., 2006; Figure 1). Among these sequences, the sequence motif FP/RK was identified as the most important with respect to the efficient targeting of proteins to the chloroplasts (Figure 1). Furthermore, this motif has been established to be fully functional in sequence contexts other than that of RbcS transit peptides (Lee et al., 2015), and has been shown to facilitate the import of preproteins with less-efficient transit peptides (Lee et al., 2009; Razzak et al., 2017). Interestingly, the findings of a site-specific cross-linking approach have indicated that the segment encompassing this motif displays a strong cross-linking patterns with not only the TOC translocon, but also Tic20, which functions as a translocation channel in the TIC translocon, thereby indicating that the FP/RK motif plays an essential role in protein translocation across TOC/TIC translocons (Richardson et al., 2018; Figure 1).

Another study identified the presence of a semi-conserved motif, FGLK, to be crucial for the interaction with TOC34 (Chotewutmontri et al., 2012; Holbrook et al., 2016; Figure 1). Together with TOC159, TOC34 is assumed to function as a receptor for chloroplast transit peptides on the outer membrane. Although the RbcS transit peptide of *Chlamydomonas reinhardtii*, a unicellular lower eukaryote, also has sequence motifs partially homologous to the *Arabidopsis* FNGLK and FP/RK motifs, this algal transit peptide proved to be non-functional when examined in *Arabidopsis* protoplasts (Razzak et al., 2017). Interestingly, the restoration of *Arabidopsis* FNGLK and FP/RK motifs in the *Chlamydomonas* RbcS transit peptide was found to markedly enhance the delivery of GFP (green fluorescent protein) to chloroplasts in *Arabidopsis* protoplasts (Razzak et al., 2017). Moreover, a synthetic transit peptide, in which the FNGLK and FP/RK motifs of the *Arabidopsis* RbcS transit peptide were incorporated, was demonstrated to deliver a vacuolar protein to chloroplasts, thereby confirming the importance of these motifs in chloroplast protein targeting (Lee et al., 2015).

A further important feature of the RbcS transit peptide sequence is its N-terminal hydrophobicity. The N-terminal region of transit peptides is characterized by a higher proportion of hydroxylated amino acids, such as serine or threonine, than that of hydrophobic amino acids (Zybailov et al., 2008). However, it has been found that when hydroxylated residues in the N-terminal region of the RbcS transit peptide are substituted with alanines, there is no corresponding perturbation of protein import into chloroplasts (Lee et al., 2006). Contrastingly, hydrophobic residues in the N-terminal region of the RbcS transit peptide (MLM motif) have been established to be essential for protein targeting from the cytosol to the chloroplasts (Lee et al., 2006; Figure 1). Moreover, this region has also been proposed to function in preprotein translocation across the chloroplast membranes by interacting with stromal Hsp70, an ATP-driven molecular motor protein (Chotewutmontri and Bruce, 2015; Figure 1).

Collectively, we have accumulated considerable knowledge regarding the organization of sequence motifs in RbcS transit peptides, which will help in elucidating the import mechanisms of chloroplast proteins and in designing more efficient transit peptides to deliver proteins into the chloroplast.

## Sequence Features Required for Chloroplast-Specific Targeting

The mitochondrion, another example of an endosymbiotic organelle, is universally present in all eukaryotic cells and is believed to have evolved prior to the chloroplast (Dyall et al., 2004). Interestingly, the protein import mechanisms of these two endosymbiotic organelles are remarkably similar, implying that plant cells possess appropriate sorting mechanisms that facilitate the site-specific targeting of proteins to chloroplasts and mitochondria (Schleiff and Becker, 2011; Lee and Hwang, 2021). A recent study accordingly sought to elucidate the putative mechanisms underlying the specificity of chloroplastic and mitochondrial protein targeting, by constructing several hybrid targeting signals consisting of segments derived from both chloroplast transit peptides and mitochondrial presequences (Lee et al., 2019). On the basis of the observed import behaviors of GFP-fused hybrid targeting signals, a simple but sophisticated principle has been deduced. Both transit peptides and presequences comprise two domains, one of which is the N-terminal specificity domain, which determines specific targeting to chloroplasts or mitochondria, and the other is the C-terminal translocation domain, which is interchangeable between the transit peptide and presequence (Lee et al., 2019; Lee and Hwang, 2021).

**Figure 2:**
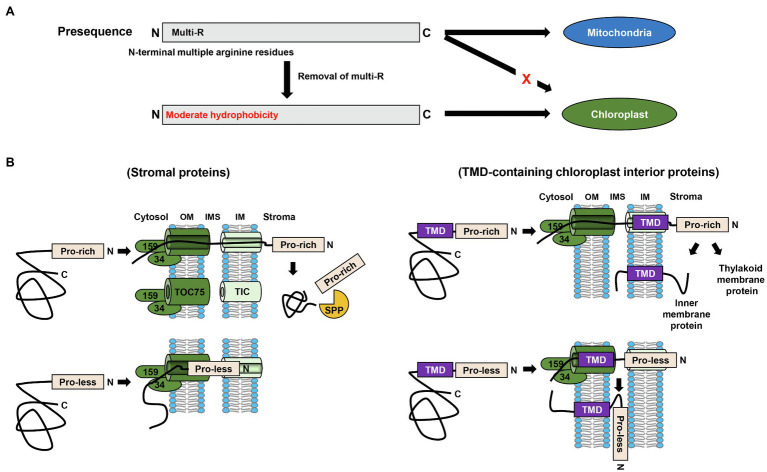
Common characteristics of different transit peptides. **(A)** Multiple arginine residues in the N-terminal region of targeting signals function as a chloroplast evasion signal. Removal of the arginine residues from the N-terminal region of mitochondrial presequences is sufficient to switch the final destination of proteins from the mitochondria to chloroplasts. **(B)** Multiple proline residues in transit peptides are essential for preprotein translocation into chloroplasts. In the case of soluble stromal proteins, preproteins with proline-less transit peptides are trapped within the TOC/TIC translocons, whereas for transmembrane domain (TMD)-containing chloroplast internal proteins, the preproteins are laterally inserted into the chloroplast outer membrane when the preproteins harbor proline-less transit peptides. TP, transit peptide; OM, outer membrane; IMS, intermembrane space; IM, inner membrane; TMD, transmembrane domain.

With regard to the common features of the N-terminal specificity domain of transit peptides, as stated in the previous section, the moderate hydrophobicity of the N-terminal region of transit peptides is critical for efficient protein import into chloroplasts. Contrastingly, however, the findings of numerous previous studies have indicated that the N-terminal region of mitochondrial presequences contains multiple arginine residues (Bhushan et al., 2006; Huang et al., 2009; Ge et al., 2014; Garg and Gould, 2016; Lee et al., 2020; Figure 2A). Surprisingly, the removal of these multiple arginine residues was sufficient to switch the final destination of cargo proteins from mitochondria to chloroplasts (Lee et al., 2019, 2020; Lee and Hwang, 2021; Figure 2A). Moreover, although mitochondrial presequences obtained from fungi and humans, which lack chloroplasts, have no need of a mechanism that discriminates between chloroplast and mitochondrial proteins, they could deliver GFP to chloroplasts when multiple arginines in the N-terminal region were substituted with alanines (Lee et al., 2020). These observations clearly indicate that the multiple arginine residues in mitochondrial presequences play a vital role in evading chloroplast targeting.

## The Roles of Proline and Basic Amino Acid Residues Commonly Present in Diverse Transit Peptides

Despite the considerable diversity among the primary structures of transit peptides, there are certain amino acids that are preferentially incorporated into the transit peptides. Among these, the roles of multiple proline residues (which are commonly present in transit peptides) in chloroplast targeting have recently been investigated (Lee et al., 2018). Proline is a unique amino acid in that it breaks the local secondary structure, thereby conferring an unstructured property to polypeptides such as transit peptides (Guzzo, 1965). Interestingly, whereas chloroplast preproteins containing proline-less transit peptides show no defects in early cytosolic steps, their translocation into chloroplasts is typically compromised (Lee et al., 2018). In this regard, the fate of unimported preproteins harboring proline-less mutant transit peptides has been found to differ depending on the characteristics of preproteins. In the case of soluble stromal proteins, unimported precursors have been found to be trapped within the TOC/TIC translocons (Figure 2B). Contrastingly, preproteins harboring hydrophobic transmembrane domain(s), such as Tic110 and Tha4 (thylakoid assembly-4), and thus destined for the inner membrane and thylakoid membrane, respectively, have been found to be integrated into the outer membrane when the transit peptides lacked proline residues (Lee et al., 2018; Figure 2B). This aberrant insertion appears to occur during preprotein translocation through the Toc75 channel *via* lateral insertion, as opposed to direct insertion from the cytosol. Notably, it was found that in the wild-type cells, the behavior of Tha4 with a proline-less transit peptide was practically similar to that of Tha4 with a proline-rich transit peptide in *hsp93-V* mutant cells (Lee et al., 2018). Given that the translocation of preproteins is compromised in the *hsp93-V* mutant, these observations provide further evidence in support of the crucial role of proline residues in preprotein translocation (Lee et al., 2015, 2018; Huang et al., 2016).

Unlike the TOM/TIM (translocase of the outer/inner membrane) translocons in the mitochondria, TOC/TIC translocons in the chloroplasts can translocate small fully folded preproteins (Ganesan et al., 2018). Even a precursor with aggregation-prone GFP[V29A], a variant of GFP, was observed to be efficiently imported into chloroplasts, which was mediated by proline-rich transit peptides (Lee et al., 2018). However, the GFP[V29A] harboring a proline-less transit peptide was found to be aggregated to a greater extent, thereby markedly compromising translocation (Lee et al., 2018). Collectively, the aforementioned observations provide compelling evidence to indicate the essential roles of multiple proline residues in transit peptides, with respect to efficient translocation of chloroplast proteins, particularly hydrophobic TMD-containing or aggregation-prone proteins.

A further common feature of different transit peptides is their preference for positively charged basic residues over negatively charged acidic residues (Bhushan et al., 2006; Zybailov et al., 2008). Indeed, it has been shown that substitution of basic residues in some transit peptides with acidic residues markedly disrupts protein import into chloroplasts (Razzak et al., 2017; Lee and Hwang, 2019). Other studies have demonstrated that a sequence motif, consisting of two consecutive basic residues, and thus referred to as a twin-positive motif, play a role in specifically facilitating the import of plastid preproteins into leucoplasts, another type of plastid (Teng et al., 2012; Chu et al., 2020). Furthermore, recent *in silico* analysis of 1,153 *Arabidopsis* plastid proteins revealed that the presence of these twin-positive motifs is significantly correlated with a higher protein abundance in root leucoplasts (Chu et al., 2020).

## Conclusion and Future Perspectives

We have now begun to understand the complex nature of the diverse range of chloroplast transit peptides. Transit peptides may have been generated *via* the assembly of distinct sequence motifs, each playing a pivotal role during interactions with the components of the TOC/TIC translocons, either sequentially or in concert. In addition to the specific short sequence motifs, there are several common sequence elements that make an essential contribution to the functionality of transit peptides. These include a lack of arginine residues in the N-terminal region, the presence of multiple proline residues, and the preferential usage of positively charged basic residues. In the future, it will be necessary to investigate how these common features coordinate the interactions of preproteins with the TOC/TIC translocons to facilitate the efficient import of proteins into chloroplasts.

## Author Contributions

DL and IH conceived this review article. JJ, DL, and IH participated in the writing of the manuscript. JJ prepared the figures. All authors contributed to the article and approved the submitted version.

## Funding

DL has been supported by an NRF grant funded by the MSIT (grant NRF-2020R1A2C4002294) and a grant from Chonnam National University (2021–2118). IH has been supported by a National Research Foundation of Korea (NRF) grant funded by the Korean government (MSIT; No. 2019R1A2B5B03099982).

## Conflict of Interest

The authors declare that the research was conducted in the absence of any commercial or financial relationships that could be construed as a potential conflict of interest.

## Publisher’s Note

All claims expressed in this article are solely those of the authors and do not necessarily represent those of their affiliated organizations, or those of the publisher, the editors and the reviewers. Any product that may be evaluated in this article, or claim that may be made by its manufacturer, is not guaranteed or endorsed by the publisher.
